# A Rare Case of Cystic Angiomatosis Involving the Bones: Our Experience and Review of Literature

**DOI:** 10.1055/s-0044-1801385

**Published:** 2025-01-09

**Authors:** Priyatesh Chandra Dwivedi, Chaitanya Borde, Ramesh Venkata Yasam, Raj Nagarkar

**Affiliations:** 1 Department of Hematology, HCG Manavata Cancer Centre, Nashik, Maharashtra, India; 2 Department of Nuclear Medicine, HCG Manavata Cancer Centre, Nashik, Maharashtra, India; 3 Department of Academics, HCG Manavata Cancer Centre, Nashik, Maharashtra, India; 4 Department of Surgical Oncology, HCG Manavata Cancer Centre, Nashik, Maharashtra, India

**Keywords:** cystic angiomatosis, metastatic malignant lesions, bone lesions, PET-CT, case report

## Abstract

Cystic angiomatosis (CA) is a rare, benign, multifocal disorder of mixed lesions (lytic, sclerotic) developed due to maldeveloped lymphatic and vascular systems. In majority of cases, it is identified fortuitously and mimicked as metastatic malignant lesions. In such cases, differential diagnosis followed by treatment is identified to be critical for stabilizing the disease and its prognosis. We reported a case of a 24-year-old female who presented to our center with complaints of lower back ache (∼4–6 weeks), intermittent pain in the abdomen, and occasional dyspnea on exertion. Imaging studies revealed mild lytic lesions and small-sized cysts in the spine, bones, and spleen. Investigations confirmed CA and follow-up treatment was given using zoledronic acid (4 mg/intravenous) every 3 months to stabilize the bone lesions. CA is a heterogeneous disease with unpredictable progression and uncertain treatment. So, it is highly recommended to disseminate research findings of all such rare cases to find any new pathophysiological findings and establish an effective treatment.

## Introduction

Cystic angiomatosis (CA) is a rare and benign pathological entity with uncertain etiology. 1 2 It is characterized by multifocal bony lesions affecting the axial and appendicular skeleton, with possible visceral organ involvement. 3 CA is predominant in males and usually discovered at the age of puberty with a second peak after 60 years. 4 Radiological imaging of this condition often resembles metastatic malignant lesions and differential diagnosis is the key to differentiate it from other conditions. Treatment is symptomatic and often a conservative approach appeared to be an appropriate option. In the majority of patients, the disease is nonprogressive and outcomes are unpredictable. Due to the rarity of the condition, literature related to this condition is limited. Given this and to understand the condition better concerning differential diagnosis, here we present a case of CA with bone lesions mimicking metastatic cancer.

## Case Report

**Fig. 1:**
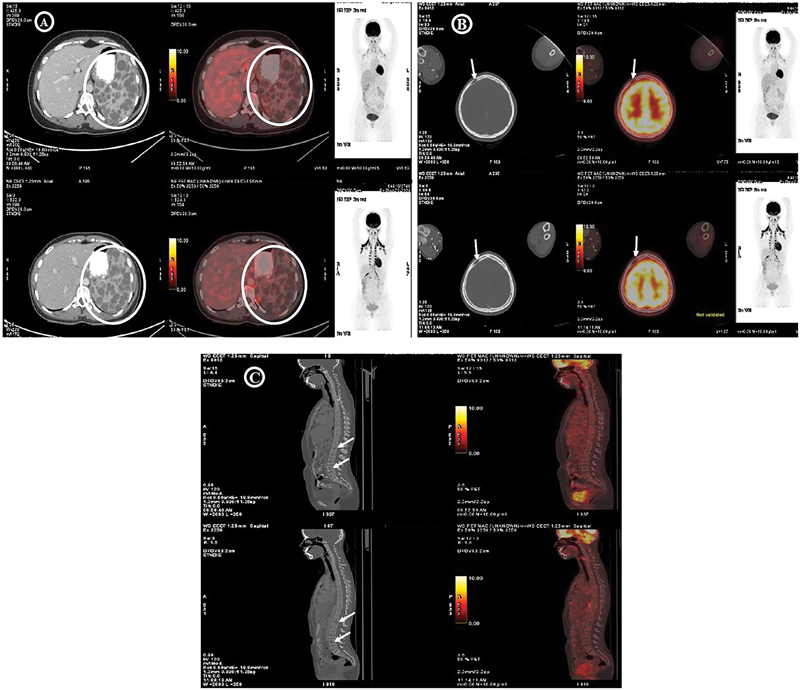
PET-CT scan. ( **A** ) Splenomegaly noted with extensive FDG non-avid cyst studded in spleen. ( **B** ) FDG non-avid, lytic lesion involving right fronto-parietal bone was noted. ( **C** ) Multiple well-defined cystic/lytic lesions involving axial skeleton are all FDG non-avid. FDG, fluorodeoxyglucose; PET-CT, positron emission tomography-computed tomography.

A 24-year-old female presented to our center with complaints of lower backache (∼4–6 weeks), intermittent pain in the abdomen, and occasional dyspnea on exertion. Ultrasound showed mild splenomegaly with multiple small-sized cysts measuring 6 to 9 mm. Computed tomography (CT) abdomen revealed multiple mild lytic lesions in the dorso-lumbar vertebrae, sacrum, and both the iliac bones. Mild splenomegaly was observed measuring 158 mm in span. Multiple varying sized cysts were reported in splenic parenchyma, with the largest measuring 33 × 27 mm. The possibility of CA or multiple myeloma was inferred. To confirm the condition, further immunofixation panel and whole-body positron emission tomography-CT (PET-CT) scan were performed. The immunofixation panel has shown no notable values. However, PET-CT has reported splenomegaly with extensive FDG (fluorodeoxyglucose) non-avid cysts studded in the spleen ( Fig. 1A ). A well-defined, FDG non-avid, cystic/lytic lesions involving the right frontoparietal bone and axial skeleton was also noted ( Fig. 1B, C ). Except for it, no other FDG avid lesions were reported, eliminating the possibility of primary or distant metastasis ( Fig. 1 ). The patient was advised for bone marrow aspiration and biopsy to complete the diagnostic investigation, but the patient refused to undergo any further tests. Differential diagnosis and reports from the tests are suggestive of CA. As a supportive treatment, the patient was started on zoledronic acid (4 mg/intravenous [IV]) every 3 months to stabilize the bone lesions. Along with zoledronic acid, cholecalciferol, calcium, and vitamin D3 oral supplementation were provided. The patient is on follow-up and regular PET-CT has shown no significant changes/new lesions in the past 2 years.

## Discussion

CA was first reported by Parsons and Ebbs 5 in the 1940s and later by Jacobs and Kimmelstiel 6 in the early 1960s. Literature reiterated CA as a benign disease resembling malignant lesions with a progressive course. In the present case report, the patient was young and female. Literature suggests that CA is generally presented in the first few decades of life, as reported in our patient. However, in some patients, its onset was reported in their 60s and it was considered as a second frequency peak. 1 3 4 CA is predominant in males over females with a ratio of 3:2. 4 CA is asymptomatic in its early stages but with its progression, the clinical symptoms such as pain in joints/spine, swelling, splenomegaly, scoliosis, and loss of strength were prominently noted. 1

Differential diagnosis of CA based on the clinical presentation itself is next to impossible. CA shares features with Gorham–Stout disease (disappearing or vanishing bone disease) and its multiple bone involvement also mimics multiple conditions such as histiocytosis, Paget's disease, multiple myeloma, lymphoma, and bone metastases. 1 7 So, radiological imaging is the only option best advised to identify the typical features of CA. The classical imaging features of CA as per literature are it should be multifocal intramedullary skeletal cysts, oriented along the long axis of the bone, and classically surrounded with a sclerotic peripheral ring with relatively well-preserved cortical bone showing no periosteal reaction. CA is also a concomitant disease and on imaging, involvement of multiple sites may be noted fortuitously. The most commonly affected sites are bone, especially of femur (∼80%), and pelvis (∼73%). 3 4 7 Visceral involvement is reported in one-fourth of cases, especially spleen (∼25% of cases), followed by skin, soft tissues, lungs, kidneys, etc. 1 2 3 4 7 The least expected sites are the tarsus, phalange, metacarpal, and carpal bones. 4 As reported in the literature, in our patient also we observed splenomegaly with multiple mild lytic lesions in the dorso-lumbar vertebrae, sacrum, and iliac bones. To date, no specific biological features have been reported related to CA. In recent times, some authors have reported to observe alkaline phosphatase levels and cell-mediated immunity (CD4 and CD8 T-cell lymphopenia). 4

Post-imaging, biopsy from bone lesions or visceral lesions can be considered for histopathological confirmation. In conditions like CA, histological diagnosis by performing a single biopsy is often unrevealing and demands multiple bone biopsies to provide a concrete diagnosis of the condition. On histopathology, the majority of the studies revealed the tissue with CA will appear to have cystic walls with flattened, single layer of endothelial cells. Furthermore, a significant proportion of the cellular structures displayed vascular channels, cavities, spaces, canals, cysts, as well as features characteristic of lymphangiomas and hemangiomas. 1 3 4 In our case, as the patient was unwilling to undergo a biopsy, CA was inferred based on imaging findings and negative immunofixation panel, which ruled out multiple myeloma.

Treatment related to CA is always supportive and it is highly dependent on the symptoms. At present, the reported treatment modalities from the literature include radiation, surgery, interferon, steroids, calcitonin, propranolol, anti-VEGF (vascular endothelial growth factor) or anti-VEGFR, and bisphosphonates. 8

Considering all the treatment options, our patient was initiated on a preventive treatment strategy, where we started our patient on zoledronic acid, 4 mg once every 6 months. Zoledronic acid has been reported to modulate and control the migration/adhesion of endothelial cells and the expression of angiogenic cytokines, which may probably stabilize the bone lesions and prevent any skeletal events. Further, Najm et al 4 have reported the use of zoledronic acid and it resulted in stabilization of bone lesions even after 2 to 4 years of use. In another study Marcucci et al, 9 an amino bisphosphonate therapy (pamidronate—30 mg IV every month) was adopted initially for 2 years followed by zoledronic acid (5 mg/once a year) for the next 3 to 5 years. At the end of the second year, improvement in bone mineral density was observed but not up to the mark and such values are still compatible with osteoporosis. However, no significant changes in the extent and size of the lytic areas were noted. By the end of 5 to 7 years, a marked improvement in densitometric values with stable bone lytic lesions was noted. 9 Given the extensive bone lesions in our patient, we decided to treat our patient using zoledronic acid alone with complementary vitamin supplementation. To date, the disease is stable and no new lesions have been noted. At the last follow-up visit, the patient showed no signs of new symptoms and is set to return for follow-up evaluations every 6 months. Inj. zoledronic acid was stopped after 2 years. From our observation and clinical experience, we conclude that CA prognosis and clinical evolution are strictly dependent on the extent of the disease and its severity, followed by its treatment strategy.

## Conclusion

Differential diagnosis of CA using various diagnostic techniques (radiological, pathological, and laboratory investigations) at the earliest is highly recommended to rule out the malignancy at an early stage to establish an accurate diagnosis. CA is also a heterogeneous, self-limiting disease of unpredictable progression and uncertain treatment, where often a conservative approach appears to be an appropriate option. In the future, it is highly recommended to disseminate research findings of all such CA rare cases to find out any new pathophysiological findings and to establish an effective treatment.
